# Supplementary material for: Forecasting biodiversity in breeding birds using best practices
Source: PeerJ. 2018 Feb 8;6:e4278. doi: 10.7717/peerj.4278 (PMC5808145; doi:10.7717/peerj.4278)
Supplement: Table S1 [file peerj-06-4278-s001.docx]

Modeling Groups and their Terms of Use

Output from yellow highlighted models is available for unrestricted use. Output from the others may only be used for non-commercial research and educational purposes. [See complete “Terms of Use”: http://cmip-pcmdi.llnl.gov/cmip5/terms.html]

| **Modeling Center (or Group)** | **Institute ID** | **Model Name** |
| --- | --- | --- |
| Beijing Climate Center, China Meteorological Administration | BCC | BCC-CSM1.1  BCC-CSM1.1(m) |
| University of Miami - RSMAS | RSMAS | CCSM4(RSMAS)* |
| National Center for Atmospheric Research | NCAR | CCSM4 |
| Community Earth System Model Contributors | NSF-DOE-NCAR | CESM1(CAM5) |
| Commonwealth Scientific and Industrial Research Organization in collaboration with Queensland Climate Change Centre of Excellence | CSIRO-QCCCE | CSIRO-Mk3.6.0 |
| The First Institute of Oceanography, SOA, China | FIO | FIO-ESM |
| NOAA Geophysical Fluid Dynamics Laboratory | NOAA GFDL | GFDL-CM3  GFDL-ESM2G  GFDL-ESM2M |
| NASA Goddard Institute for Space Studies | NASA GISS | GISS-E2-R |
| National Institute of Meteorological Research/Korea Meteorological Administration | NIMR/KMA | HadGEM2-AO |
| Met Office Hadley Centre (additional HadGEM2-ES realizations contributed by Instituto Nacional de Pesquisas Espaciais) | MOHC  (additional realizations by INPE) | HadGEM2-ES |
| Institut Pierre-Simon Laplace | IPSL | IPSL-CM5A-LR  IPSL-CM5A-MR |
| Japan Agency for Marine-Earth Science and Technology, Atmosphere and Ocean Research Institute (The University of Tokyo), and National Institute for Environmental Studies | MIROC | MIROC-ESM  MIROC-ESM-CHEM |
| Atmosphere and Ocean Research Institute (The University of Tokyo), National Institute for Environmental Studies, and Japan Agency for Marine-Earth Science and Technology | MIROC | MIROC5 |
| Norwegian Climate Centre | NCC | NorESM1-M  NorESM1-ME |
