# Supplementary material for: Forecasting biodiversity in breeding birds using best practices
Source: PeerJ. 2018 Feb 8;6:e4278. doi: 10.7717/peerj.4278 (PMC5808145; doi:10.7717/peerj.4278)
Supplement: Supplemental Information 1 [file peerj-06-4278-s002.pdf]

Supplement to "Forecasting biodiversity in breeding birds using best practices"

by David J. Harris, Shawn D. Taylor, and Ethan P. White

Observer model description

Richness at site i, as recorded by observer j is estimated using a linear mixed model. Thus, the response variable was modeled as $y_{ij} \sim \mathcal{N}(\mu_{ij}, \sigma^{\text{residual}})$.

Here, μ_{ij} is defined as an intercept term, plus a site-level effect, plus an observer-level effect: $\mu_{ij} = \alpha + \alpha_i^{\text{site}} + \alpha_j^{\text{observer}}$.

These two effects are each drawn from zero-mean Gaussians: $\alpha^{\text{site}} \sim \mathcal{N}(0, \sigma^{\text{site}})$ and $\alpha^{\text{observer}} \sim \mathcal{N}(0, \sigma^{\text{observer}})$.

Prior distributions on α, σ^{residual}, σ^{site}, and σ^{observer} are included below.

Stan code

```
data {
  int N;
  int N_site;
  int N_observer;
  int N_test_observer;
  int site_index[N];
  int observer_index[N];
  real richness[N];
}
parameters {
  vector[N_site] site_effect;
  vector[N_observer] observer_effect;
  real intercept;
  real<lower=0> site_sigma;
  real<lower=0> observer_sigma;
  real<lower=0> sigma;
}
model {
  // priors
  intercept ~ normal(mean(richness), 5 * sd(richness));

  site_sigma ~ normal(0, sd(richness));
  observer_sigma ~ normal(0, sd(richness));
  sigma ~ normal(0, sd(richness));

  // Latent variables
  site_effect ~ normal(0, site_sigma);
  observer_effect ~ normal(0, observer_sigma);

  // observation model
  richness ~ normal(
    intercept + site_effect[site_index] + observer_effect[observer_index],
    sigma
  );
}
generated quantities {
  vector[N_test_observer] test_observer_effect;
  for (i in 1:N_test_observer) {
    test_observer_effect[i] = normal_rng(0, observer_sigma);
  }
}
```

Details of the auto.arima model

We used non-seasonal time series model $ARIMA(p, d, q)$, with drift allowed, in the function `auto.arima` of the R package `forecast`. The search space for p and q were $(2, 5)$ and $(2, 5)$, respectively. d was selected via KPSS unit-root tests as described in Hyndman and Khandakar (2008) and with a maximum value of 2. Stepwise model selection was done using AICc as the model criteria.
